# Supplementary material for: A Likelihood Approach for Real-Time Calibration of Stochastic Compartmental Epidemic Models
Source: PLoS Comput Biol. 2017 Jan 17;13(1):e1005257. doi: 10.1371/journal.pcbi.1005257 (PMC5240920; doi:10.1371/journal.pcbi.1005257)
Supplement: S1 File — (TAR.GZ) [file pcbi.1005257.s014.tar.gz › HSPH_Online-SI-Revision/MSS10/n1-mild/n1-mild_NEW_table-4.pdf]

1	R0	{1.27677, 7.89375, 1.}	{1.56707, 11.0367, 0.72}	{{1.38427, 6.71734, 0.}, {1.95665, 16.748, 2.}}	{23.1615, 45.3642, 60.}	inf	17.	30.2857	{13.1447, 48.2502}	87.1567	0.253088	newly	24.	21.63	{11., 32.}	29.4583	0.152115	Reff	1.16697	1.40868	{1.25251, 1.73903}	21.1091	ar	400.	566.29	{381., 790.}	50.0375
2	R0	{1.33231, 10.5259, 2.}	{1.26067, 4.79921, 1.26}	{{1.24271, 3.26445, 0.}, {1.28596, 5.50828, 2.}}	{5.39558, 54.4057, 37.}	inf	39.	18.6904	{4.63615, 28.9529}	52.2934	0.393879	newly	28.	25.4	{9., 45.}	40.9286	0.233543	Reff	0.927288	0.903031	{0.879761, 0.927672}	2.92211	ar	467.	370.85	{291., 456.}	21.2741
3	R0	{1.2273, 6.76944, 1.}	{1.14001, 2.40389, 0.02}	{{1.06738, 2.19254, 0.}, {1.24751, 3.07157, 0.}}	{8.83102, 64.4891, 98.}	inf	19.	16.468	{3.32589, 33.6876}	57.6882	0.337598	newly	22.	50.78	{6., 116.}	163.	0.409766	Reff	1.11071	1.03408	{0.965849, 1.13907}	8.57325	ar	314.	247.46	{84., 438.}	40.4459
4	R0	{1.41215, 17.5742, 3.}	{1.15143, 3.70613, 0.46}	{{1.06738, 2.19254, 0.}, {1.34829, 9.33397, 2.}}	{18.8687, 79.0948, 86.}	inf	31.	14.1859	{3.34625, 28.521}	62.1683	0.511838	newly	15.	29.42	{7., 61.}	115.333	0.320935	Reff	1.27376	1.05693	{0.978673, 1.22688}	17.2714	ar	465.	245.21	{76., 483.}	51.6237
5	R0	{1.42222, 6.15886, 0.1}	{1.17811, 2.90551, 0.17}	{{1.06738, 2.19254, 0.}, {1.25474, 3.26445, 1.}}	{17.1638, 52.8239, 240.}	inf	27.	10.8444	{3.44254, 19.4318}	60.5762	0.486664	newly	31.	26.51	{6., 52.}	46.8065	0.29201	Reff	1.21173	1.02279	{0.9252, 1.09739}	15.5929	ar	490.	230.92	{131., 380.}	52.8735
6	R0	{1.24363, 9.95596, 0.1}	{1.48159, 11.4207, 1.08}	{{1.10202, 5.30671, 0.}, {1.99753, 18.8228, 2.}}	{25.8182, 50.0824, 1046.}	inf	12.	6.35476	{1.69678, 12.4144}	52.3882	0.400895	newly	7.	5.23	{1., 8.}	60.4286	0.420371	Reff	1.21503	1.45495	{1.08869, 1.93239}	25.9442	ar	411.	402.06	{15., 798.}	71.4453
7	R0	{1.36478, 5.96056, 1.}	{1.63359, 7.75464, 1.83}	{{1.39162, 1.89883, 0.}, {1.99485, 17.6755, 5.}}	{19.7004, 107.327, 199.}	inf	11.	64.6697	{17.2125, 115.077}	494.452	0.697094	newly	13.	121.08	{32., 282.}	834.462	0.831307	Reff	1.2092	1.35052	{1.14421, 1.58232}	14.1672	ar	484.	615.51	{415., 829.}	37.1756
8	R0	{1.36335, 17.6429, 2.}	{1.29507, 16.4034, 0.89}	{{1.12882, 6.71734, 0.}, {1.34361, 18.7523, 1.}}	{5.23713, 16.3316, 55.5}	inf	30.	24.6518	{7.5436, 34.7316}	26.169	0.175876	newly	14.	10.8	{5., 16.}	35.8571	0.218782	Reff	1.27473	1.21706	{1.07649, 1.26722}	4.84044	ar	484.	428.83	{182., 566.}	22.6591
9	R0	{1.26575, 5.28707, 0.1}	{1.24825, 3.53721, 0.16}	{{1.21159, 3.07157, 0.}, {1.28596, 3.98513, 1.}}	{2.97887, 33.097, 232.}	inf	13.	12.6034	{3.73225, 21.1241}	48.7746	0.256487	newly	20.	26.89	{6., 51.}	70.95	0.291304	Reff	1.05943	1.04526	{1.00961, 1.08465}	3.0427	ar	472.	307.58	{165., 456.}	35.7627
10	R0	{1.42942, 9.49905, 2.}	{1.54369, 13.1866, 2.26}	{{1.34436, 6.58803, 0.}, {1.6004, 16.157, 3.}}	{10.461, 51.1384, 40.}	inf	45.	70.5546	{23.6805, 102.464}	76.9242	0.269503	newly	34.	37.49	{21., 51.}	28.6765	0.131765	Reff	1.07635	1.11775	{1.02116, 1.1564}	5.76937	ar	468.	586.83	{386., 743.}	33.1261
11	R0	{1.46827, 15.0983, 0.1}	{1.1254, 10.6079, 1.72}	{{1.10133, 5.30671, 0.}, {1.17082, 14.812, 3.}}	{23.3518, 30.2591, 1684.}	inf	28.	8.25323	{2.38028, 12.5158}	71.0641	0.614692	newly	14.	6.48	{1., 13.}	57.5714	0.485436	Reff	1.36843	1.07109	{1.04444, 1.1212}	21.7468	ar	495.	165.54	{48., 341.}	67.1717
12	R0	{1.25751, 13.279, 1.}	{1.14567, 13.103, 3.43}	{{1.13227, 5.71232, 0.}, {1.1546, 14.5539, 4.}}	{8.89363, 17.4546, 267.}	inf	18.	9.27369	{4.15227, 13.0297}	48.4795	0.331943	newly	8.	5.36	{2., 8.}	43.25	0.291279	Reff	1.17829	1.08344	{1.07811, 1.09445}	8.04959	ar	366.	198.35	{63., 357.}	46.4781
13	R0	{1.3425, 9.16361, 1.}	{1.29104, 4.9842, 0.87}	{{1.22544, 3.07157, 0.}, {1.31748, 5.50828, 2.}}	{6.59589, 56.0579, 117.}	inf	32.	21.1788	{5.59477, 35.1334}	52.3551	0.352596	newly	28.	28.18	{7., 48.}	43.1429	0.230403	Reff	0.965258	0.940827	{0.89638, 0.969117}	4.45209	ar	442.	372.95	{268., 465.}	22.6719
14	R0	{1.18936, 6.20583, 0.1}	{1.56274, 8.72738, 0.94}	{{1.06738, 2.19254, 0.}, {2.29738, 19.0355, 2.}}	{34.8582, 87.1304, 944.}	inf	26.	47.5782	{5.50476, 109.362}	127.512	0.408767	newly	32.	40.83	{12., 65.}	53.1563	0.216542	Reff	1.01333	1.2825	{0.929712, 1.76585}	29.6237	ar	382.	507.62	{169., 848.}	62.1204
15	R0	{1.27137, 9.78868, 0.1}	{1.25418, 4.05251, 0.41}	{{1.08063, 2.5364, 0.}, {1.28596, 3.98513, 1.}}	{5.98106, 65.8036, 464.}	inf	27.	18.3251	{4.39297, 33.9165}	55.725	0.376386	newly	24.	34.53	{9., 62.}	74.2917	0.269771	Reff	1.10101	1.09556	{0.949555, 1.14483}	5.24524	ar	496.	323.21	{127., 477.}	39.5625
16	R0	{1.23102, 13.6452, 1.}	{1.73591, 14.1825, 1.12}	{{1.39882, 8.35044, 0.}, {1.95665, 16.748, 3.}}	{41.014, 26.6092, 42.}	inf	33.	61.2916	{28.339, 85.8486}	92.958	0.271082	newly	16.	33.31	{22., 42.}	109.063	0.306592	Reff	1.07222	1.45797	{1.22166, 1.63511}	35.9768	ar	445.	681.25	{510., 800.}	53.8045
17	R0	{1.27213, 13.7452, 3.}	{1.58518, 11.8591, 0.75}	{{1.13726, 5.77111, 0.}, {1.95665, 18.8228, 2.}}	{27.8008, 32.4726, 75.}	inf	22.	19.7236	{6.82632, 33.1738}	41.5651	0.222952	newly	11.	13.53	{5., 23.}	49.3636	0.202669	Reff	1.19962	1.49573	{1.08814, 1.82325}	27.5228	ar	367.	562.58	{115., 794.}	74.8174
18	R0	{1.25318, 11.2151, 0.1}	{1.24453, 14.2678, 3.97}	{{1.23973, 14.2527, 4.}, {1.23973, 14.2527, 4.}}	{1.39199, 27.2198, 3870.}	inf	26.	29.1521	{23.9304, 34.9658}	19.581	0.0847851	newly	13.	14.69	{10., 20.}	26.2308	0.112586	Reff	1.10029	1.08887	{1.07733, 1.09059}	1.87746	ar	416.	396.11	{274., 484.}	15.3678
19	R0	{1.46006, 8.11496, 0.1}	{2.38565, 15.0915, 0.2}	{{2.11132, 12.644, 0.}, {2.81891, 18.2812, 1.}}	{64.152, 90.2, 266.}	inf	42.	120.686	{83.6215, 160.311}	190.194	0.453046	newly	40.	65.58	{49., 81.}	65.05	0.211779	Reff	1.24251	1.83279	{1.67921, 2.07259}	48.1719	ar	496.	853.82	{807., 923.}	73.746
20	R0	{1.14273, 6.71261, 0.1}	{1.30838, 5.89476, 0.69}	{{1.08063, 2.5364, 0.}, {1.6004, 16.157, 3.}}	{16.0176, 57.7376, 728.}	inf	15.	23.918	{4.31056, 43.1165}	87.7216	0.312313	newly	12.	35.94	{7., 66.}	213.333	0.472337	Reff	1.02046	1.15533	{0.967796, 1.35709}	14.704	ar	362.	384.67	{119., 604.}	38.0691
21	R0	{1.42432, 8.23905, 1.}	{1.12628, 2.46379, 0.05}	{{1.06738, 2.19254, 0.}, {1.24751, 3.07157, 0.}}	{20.9252, 70.0962, 99.}	inf	17.	5.93939	{1.85004, 11.1827}	65.2999	0.543754	newly	14.	16.06	{3., 32.}	64.5714	0.304439	Reff	1.15512	0.925794	{0.874042, 1.02268}	19.8532	ar	405.	220.34	{175., 272.}	45.9605
22	R0	{1.29386, 17.863, 2.}	{1.81233, 14.3743, 0.42}	{{1.38427, 8.26611, 0.}, {2.11476, 19.5406, 1.}}	{40.0715, 24.779, 79.}	inf	27.	31.7547	{11.6732, 53.0336}	47.8	0.197013	newly	8.	18.15	{9., 28.}	132.375	0.340914	Reff	1.20588	1.67656	{1.30363, 1.93346}	39.0327	ar	488.	699.73	{496., 844.}	46.4857
23	R0	{1.39274, 13.0167, 5.}	{1.17854, 3.21711, 0.24}	{{1.06738, 2.19254, 0.}, {1.28596, 3.98513, 1.}}	{15.38, 75.2847, 95.2}	inf	34.	12.5451	{3.45063, 22.7424}	63.1027	0.532139	newly	12.	26.18	{6., 50.}	139.	0.371254	Reff	1.12533	0.977263	{0.882739, 1.07186}	13.1579	ar	475.	262.64	{165., 409.}	44.8463
24	R0	{1.32178, 15.8991, 1.}	{1.647, 17.2695, 1.9}	{{1.50403, 15.3523, 2.}, {1.75814, 19.4054, 2.}}	{24.7823, 17.7688, 92.}	inf	43.	39.0596	{21.9393, 56.125}	26.8094	0.133959	newly	19.	17.06	{11., 24.}	24.9474	0.124267	Reff	1.16978	1.46254	{1.33612, 1.5607}	25.0564	ar	471.	655.44	{527., 743.}	40.3397
25	R0	{1.37568, 17.8427, 1.}	{1.42008, 14.6626, 2.44}	{{1.11523, 3.07157, 0.}, {1.48819, 17.9732, 3.}}	{10.2176, 19.4015, 168.}	inf	18.	29.8625	{9.00576, 41.6952}	83.1304	0.290735	newly	6.	17.03	{9., 26.}	184.833	0.407764	Reff	1.20647	1.22704	{0.988062, 1.29166}	8.47032	ar	398.	487.1	{189., 618.}	41.9899
26	R0	{1.20993, 6.4656, 0.1}	{1.50564, 11.904, 1.94}	{{1.38427, 8.35044, 0.}, {1.85782, 17.7016, 3.}}	{24.4401, 84.1127, 1866.}	inf	13.	48.8393	{32.2263, 63.7592}	275.825	0.546249	newly	16.	30.8	{19., 44.}	93.375	0.268462	Reff	1.03933	1.23648	{1.15526, 1.50455}	18.985	ar	345.	564.	{436., 742.}	63.8783
27	R0	{1.20548, 13.8003, 0.1}	{1.64377, 14.9652, 0.33}	{{1.514, 12.483, 0.}, {1.76949, 19.5271, 2.}}	{36.3584, 16.5343, 392.}	inf	42.	38.0473	{25.061, 56.0135}	23.6045	0.11454	newly	24.	20.03	{10., 32.}	34.125	0.182382	Reff	1.0548	1.44536	{1.29434, 1.57573}	37.0275	ar	350.	645.45	{502., 736.}	85.0657
28	R0	{1.3176, 14.6594, 1.}	{1.59623, 15.3311, 0.86}	{{1.27063, 10.8657, 0.}, {1.8951, 19.1478, 2.}}	{23.843, 20.4758, 82.}	inf	14.	11.5515	{3.20525, 24.8751}	55.6987	0.328158	newly	4.	6.09	{1., 12.}	95.75	0.394767	Reff	1.27412	1.54581	{1.23257, 1.82373}	23.7811	ar	362.	529.04	{38., 788.}	77.8066
29	R0	{1.28202, 6.23047, 1.}	{1.58611, 13.0354, 1.75}	{{1.25474, 3.57392, 0.}, {1.91377, 18.7084, 2.}}	{25.1906, 124.549, 99.}	inf	36.	50.208	{15.8373, 84.3621}	59.8869	0.228089	newly	35.	30.6	{16., 43.}	30.5714	0.156412	Reff	1.10382	1.33856	{1.11561, 1.56142}	22.3694	ar	391.	602.05	{337., 781.}	62.7442
30	R0	{1.28177, 8.83252, 1.}	{1.6875, 10.8063, 1.42}	{{1.28596, 4.00571, 0.}, {1.85253, 15.8132, 3.}}	{33.4714, 43.1354, 98.}	inf	30.	57.374	{21.7199, 80.9727}	104.46	0.312313	newly	21.	42.53	{28., 56.}	104.905	0.300135	Reff	1.12539	1.43009	{1.1545, 1.56388}	28.7177	ar	427.	655.55	{440., 781.}	59.9274
31	R0	{1.35886, 5.97708, 0.1}	{1.8843, 14.8895, 2.08}	{{1.44466, 12.0176, 2.}, {2.05052, 16.7932, 3.}}	{38.8643, 150.082, 1986.}	inf	23.	81.4326	{48.2096, 100.156}	254.055	0.528806	newly	44.	42.4	{28., 55.}	19.5455	0.090773	Reff	1.14008	1.46668	{1.18023, 1.5845}	28.8721	ar	491.	736.57	{573., 826.}	50.5356
32	R0	{1.27601, 17.9804, 3.}	{1.93073, 19.1563, 0.09}	{{1.45613, 15.876, 0.}, {1.98971, 19.6444, 0.}}	{51.3096, 9.93287, 97.}	inf	25.	41.104	{31.7573, 50.146}	65.3924	0.212333	newly	8.	17.06	{11., 23.}	113.5	0.313575	Reff	1.18924	1.76757	{1.36241, 1.83558}	48.6297	ar	445.	770.25	{623., 827.}	73.0899
33	R0	{1.3368, 11.3235, 0.1}	{1.5861, 12.5206, 1.14}	{{1.38427, 8.26611, 0.}, {1.85782, 17.7016, 2.}}	{20.6565, 38.8333, 1114.}	inf	40.	41.4659	{19.8331, 67.0102}	39.9139	0.185007	newly	21.	26.21	{16., 38.}	36.2381	0.136273	Reff	1.1697	1.38161	{1.20537, 1.60696}	19.6961	ar	417.	579.47	{336., 773.}	50.5444
34	R0	{1.21021, 11.3635, 2.}	{1.13114, 4.74532, 0.27}	{{1.08063, 3.02112, 0.}, {1.25656, 5.71232, 1.}}	{8.18312, 58.2407, 87.5}	inf	20.	9.38326	{4.22921, 15.6676}	54.0403	0.395185	newly	14.	15.71	{5., 32.}	59.2143	0.254575	Reff	1.06862	1.01075	{0.961833, 1.12644}	7.45762	ar	327.	201.17	{105., 330.}	41.422
35	R0	{1.53382, 10.8866, 0.1}	{1.98671, 16.5294, 0.59}	{{1.75663, 14.1819, 0.}, {2.22832, 19.5406, 1.}}	{30.3662, 54.0201, 578.}	inf	30.	61.8937	{46.6651, 79.1444}	108.352	0.312154	newly	22.	31.02	{21., 42.}	44.4545	0.152028	Reff	1.3789	1.72088	{1.52896, 1.88382}	25.5815	ar	476.	781.47	{708., 853.}	64.9391
36	R0	{1.20423, 6.83844, 0.1}	{2.13471, 16.5132, 1.4}	{{1.73739, 11.1369, 0.}, {2.46533, 19.5128, 2.}}	{77.2674, 141.476, 1320.}	inf	44.	79.1653	{50.1143, 105.45}	80.7325	0.243591	newly	49.	39.98	{27., 53.}	21.6735	0.114633	Reff	1.05852	1.7971	{1.5206, 2.03564}	69.7755	ar	416.	814.16	{674., 896.}	95.7115
37	R0	{1.43629, 10.1548, 3.}	{1.1786, 3.32709, 0.53}	{{1.06738, 2.19254, 0.}, {1.28596, 3.98513, 2.}}	{18.1485, 67.375, 83.}	inf	15.	12.6716	{4.67617, 24.0002}	44.1144	0.255708	newly	13.	27.86	{8., 51.}	130.923	0.338302	Reff	1.27255	1.04681	{0.947402, 1.14892}	17.9128	ar	367.	249.57	{112., 431.}	42.3351
38	R0	{1.37504, 8.93428, 1.}	{1.98844, 13.7635, 1.79}	{{1.56097, 8.77207, 0.}, {2.48947, 19.255, 2.}}	{46.0819, 62.0041, 99.}	inf	32.	83.9918	{43.8131, 128.844}	168.607	0.417843	newly	24.	47.87	{33., 62.}	102.708	0.303597	Reff	1.17703	1.58729	{1.30267, 1.8904}	36.1588	ar	483.	750.62	{604., 887.}	59.9627
39	R0	{1.51637, 9.36742, 1.}	{1.30993, 5.11285, 1.26}	{{1.24271, 3.98513, 0.}, {1.3618, 5.50828, 2.}}	{13.8133, 48.4257, 86.}	inf	43.	23.8312	{8.43738, 40.3038}	46.7121	0.336274	newly	31.	34.27	{13., 60.}	45.3226	0.209329	Reff	1.204	1.06485	{1.01709, 1.11705}	11.6929	ar	441.	388.79	{220., 501.}	20.1474
40	R0	{1.23658, 9.82869, 1.}	{1.1822, 2.88081, 0.15}	{{1.06738, 2.19254, 0.}, {1.25474, 3.57392, 1.}}	{5.80576, 70.6898, 87.}	inf	29.	11.3317	{2.28613, 22.1184}	60.9253	0.514097	newly	15.	31.07	{6., 60.}	131.8	0.365861	Reff	1.09561	1.06813	{0.9654, 1.13734}	5.62087	ar	414.	263.24	{92., 437.}	39.6715
41	R0	{1.24247, 5.4242, 0.1}	{1.94886, 13.0729, 1.95}	{{1.06738, 2.19254, 0.}, {2.39393, 19.7817, 3.}}	{59.954, 159.618, 1898.}	inf	23.	59.1864	{16.2166, 95.3666}	168.976	0.404474	newly	36.	40.45	{26., 59.}	29.9167	0.115988	Reff	1.1381	1.70184	{0.995734, 2.0582}	52.5002	ar	345.	710.16	{248., 898.}	117.287
42	R0	{1.28208, 4.40223, 0.1}	{1.76328, 14.2764, 2.44}	{{1.44762, 10.5617, 2.}, {2.05052, 18.788, 3.}}	{37.8679, 226.366, 2354.}	inf	20.	31.6822	{15.141, 47.6427}	68.1517	0.223951	newly	30.	18.81	{9., 27.}	43.3	0.273227	Reff	1.1949	1.62057	{1.35657, 1.9129}	35.9367	ar	339.	688.03	{551., 807.}	106.31
43	R0	{1.56773, 6.50474, 0.1}	{1.52952, 5.40298, 0.45}	{{1.06738, 2.19254, 0.}, {2.688, 14.8279, 2.}}	{31.2355, 70.0451, 506.}	inf	33.	51.3232	{6.75694, 142.292}	109.55	0.377035	newly	44.	68.14	{16., 120.}	82.0455	0.289441	Reff	1.34982	1.26069	{0.92471, 1.96111}	26.008	ar	498.	462.66	{189., 918.}	45.2289
44	R0	{1.10489, 6.01941, 1.}	{1.1537, 2.81368, 0.19}	{{1.06738, 2.19254, 0.}, {1.25474, 3.26445, 1.}}	{7.68246, 53.2565, 81.}	inf	12.	11.1252	{3.14648, 20.8101}	47.8938	0.260966	newly	14.	26.19	{8., 48.}	110.786	0.321903	Reff	0.942471	0.985054	{0.906018, 1.07352}	7.59311	ar	346.	250.98	{144., 400.}	34.9711
45	R0	{1.25888, 14.1926, 1.}	{1.24496, 5.70319, 1.68}	{{1.17082, 3.98513, 0.}, {1.28596, 6.0993, 2.}}	{3.43686, 60.4057, 92.}	inf	50.	20.1269	{7.58711, 31.8028}	62.6757	0.480517	newly	23.	24.38	{9., 40.}	45.3043	0.215617	Reff	0.981926	1.00793	{0.951873, 1.04829}	4.19915	ar	471.	333.43	{201., 446.}	31.6539
46	R0	{1.45292, 16.616, 2.}	{1.95004, 14.2045, 0.27}	{{1.63746, 10.1332, 0.}, {2.66225, 19.9254, 1.}}	{34.5891, 21.4641, 86.5}	inf	34.	39.5093	{21.3322, 79.1192}	39.7454	0.148867	newly	17.	23.07	{13., 33.}	46.6471	0.160846	Reff	1.34105	1.78371	{1.52131, 2.3123}	33.327	ar	494.	765.11	{663., 904.}	55.3016
47	R0	{1.22528, 16.6033, 0.1}	{1.67097, 14.9188, 0.53}	{{1.40261, 10.7271, 0.}, {1.8951, 19.1994, 1.}}	{36.4308, 17.953, 524.}	inf	14.	16.4725	{10.3098, 23.3207}	32.6096	0.122106	newly	6.	9.14	{4., 14.}	66.3333	0.216795	Reff	1.17014	1.59111	{1.33969, 1.79862}	36.018	ar	368.	654.93	{481., 785.}	78.5462
48	R0	{1.1669, 6.92715, 0.1}	{1.49222, 17.2751, 4.32}	{{1.18976, 6.0871, 0.}, {1.56495, 19.9037, 5.}}	{28.679, 157.666, 4240.}	inf	17.	51.4495	{10.69, 66.9302}	215.349	0.493749	newly	17.	21.07	{12., 28.}	35.1176	0.135574	Reff	0.977862	1.1962	{0.994145, 1.25333}	22.9635	ar	416.	551.12	{247., 673.}	47.5144
49	R0	{1.32749, 16.0887, 3.}	{1.33462, 10.6057, 0.86}	{{1.13726, 6.0871, 0.}, {1.45954, 13.8428, 2.}}	{6.7739, 37.0471, 76.}	inf	24.	12.6321	{5.54402, 20.1872}	52.0042	0.360724	newly	8.	9.27	{4., 15.}	46.625	0.199645	Reff	1.24917	1.2705	{1.09016, 1.3946}	7.12045	ar	353.	384.86	{66., 569.}	48.034
50	R0	{1.29636, 15.2609, 0.1}	{1.67529, 16.8774, 1.59}	{{1.38427, 8.35044, 0.}, {1.75814, 19.4054, 2.}}	{29.2307, 30.687, 1532.}	inf	50.	66.4495	{38.0443, 82.7332}	43.3809	0.160624	newly	20.	29.02	{20., 36.}	46.5	0.157949	Reff	1.03838	1.31207	{1.11763, 1.38006}	26.3567	ar	470.	661.09	{461., 748.}	42.3553
